# Hammer On

**Published:** 1860-06

**Authors:** 


### HAMMER ON.

FORTUNATE are they who, while they are living, can witness the fruit of their good doings, and are further rewarded by evidences of a grateful appreciation on the part of those for whose benefit they live and labor.

A worthy and conscientious clergyman—and there are many such—was greatly distressed in the inability to see that he was living to purpose: his advice was unheeded; his counsels disregarded, he seemed to himself like one who was beating the air, and that the work of his hands was labor lost. Nor was this the greater trouble; with the humility which belongs to a good heart, he began to lay all the blame on himself; to feel that he was not only not doing any good, but was hindering some other and more able laborer from saving the "lost." In such a frame of mind, scarcely knowing what to do, it is related that he had a dream, to the effect, that with the tiniest hammer, he was appointed to break in pieces an immense rock. The task seemed utterly hopeless, yet he did as he was bid, without seeing that he made the slightest impression, and he was on the very point of exclaiming, in irrepressible impatience, "Wherefore all this useless labor?" when the huge mass crumbled to pieces. He was one of that happy class of minds which endeavored habitually to see the hand of Providence in all that occurred, and felt and sang

> "In each event of life how clear,
> Thy ruling hand I see."

And with unwonted alacrity, he girded himself to new and wider labors, with the result of gathering such a harvest as in his wildest imagination he never dreamed of.

While this is a beautifully encouraging lesson to all who are called to work in darkness, it should make those thankful who are permitted to see that they are doing a good work, that they are accomplishing useful ends, and that lookers-on clap their hands and pleasantly exclaim: "Good speed be to ye."

This latter is the editor's lot in conducting the JOURNAL OF HEALTH. Letters of commendation are coming in from all quarters, written by stranger hands; names unknown take upon themselves a personal visit, to express a word of encouragement. A professional gentlemen of this city, who is destined to eminence, made free to say: "I hope you will never abandon your writing, whatever you may do as to your profession. You are filling a space which was never thus occupied before, and which is of the highest importance. I hope you will hammer on."

We do confess to an abiding yearning after earlier paths, and can not say what a day may bring forth: we make no engagements, only to let every thing be arranged for perfect freedom of action when the time for action comes. Fetters, avaunt!

A correspondent says: "There was one suggestion in one of the early numbers of 1859, which has been worth a dollar a week to me every since. I allude to your advice to eat absolutely nothing after four o'clock in the afternoon. My sleep, since I acted on this advice, has been so much sounder and more refreshing, that I begin to feel like another man.

"I was much interested in the bachelor's tract, on page 279, of vol. 6, 1859. Inclosed is something similar, a re-publication of essays to do good, selected from HALL'S JOURNAL and others, which is being circulated here gratuitously, in the hope that it may help to happify many hearts and homes.

"It is my intention to do more than ever this year to extend the knowledge of your JOURNAL among clergymen and theological students, many of whom are perishing, or leading comparatively useless lives, for want of the hints it gives, and which can be found no where else that I know of, unless where they are so mixed up with phrenology, or a quasi-infidelity, that these good men are not likely to esteem as truth what they find in such doubtful company."

Will the Messrs. Fowler, the editor of the *Herald of Progress* and the *Water Cure*, who write so many good things in reference to temperance and a wise life, make a note of it that we are only making a verbatim quotation?!

A letter dated from the "House of Representatives" says:

"I feel constrained to say, that I am more than satisfied with your JOURNAL, as eminently deserving the support of an intelligent people. Indeed, I have said more than once, 'It is worth fifty dollars a year,' and I believe it."

Another writes: "I had the opportunity, a part of last winter and spring, of reading your JOURNAL OF HEALTH, at the house of a relative, and I was so well pleased with it, that I have desired to have it myself, but I can not spare the dollar. I am an invalid, a broken-down clergyman; I have no salary, and consequently can not send you the subscription-price. I hope you will not give me the go-by, for I will pay you as soon as I am able."

Reader, don't you wish you had the chance of paying for that subscription? You can't have it; that's all. But you might do well to order the JOURNAL OF HEALTH for your own minister; it would be difficult for you to spend a dollar for him to greater advantage, or to make so acceptable a present at so little cost. We have had a number of contributions of this sort, but, with a very few exceptions, they have all come from the south of Mason & Dixon's line. Who will explain it?

A letter just received from a Southern planter, in closing an account of long years of suffering from nervousness and sudden attacks of debility, says: "When half a mile only from home, I would at times feel as if I could never get there. About that time, I began to take your JOURNAL OF HEALTH, and then obtained your book on Health and Disease, and learned how to manage myself better, and have gradually improved every year. I had no hope of getting better, but after I found I improved every year from reading your writings, (for there is no doubt it was the saving of me,) I thought I would now explain my case to you."

Letters like the above, from unknown persons, and verbal relations of strangers from a distance, who come to express their gratifications in person, are, without exaggeration, of almost daily occurrence, and are certainly calculated to encourage us to "hammer on," in cheerful hope that we are doing a substantial good to many whom we may never see or know. Let such of these as may chance to read this page, pay us in full, by doing some act of goodness to any brother being next them, with the sweet assurance of promoting human harmonies thereby, and that

"It will all be right in the morning."

A literary gentleman wrote us [illegible]ve years ago: "I read your JOURNAL with great pleasure and profit, the only medical work I ever saw that was full of one's own symptoms, and yet not calculated to give him the blues, *en avant.*" Oui, Monsieur, Ya, Mynheer, Si, Signor. In the light of these things we will "hammer on," not, however, with the happy don't-careativeness and abandon of former years; for, with a steadily-increasing circulation, there grows apace an increasing feeling of responsibility, and a fear of compromising any influence for good which the JOURNAL possesses, by some ill-advised expression or sentiment. We fear that we are approaching the confines of the other half of the century, for we do daily find that there is a beginning of "fears in the way," that the repressive influences of "policy" are more and more decidedly felt; an inkling after conciliation rather than provocation; an effort to "walk softly;" there is an instinct for whispering out unpalatable truths, and for assuming the "stereotype smile." Tell us, ye aged pilgrims on the lowering side of fifty, if it was thus with you at an earlier time? Say, is it an infallible sign of coming age? If so, please remember all signs fail in dry weather. Our family Bible was lost in a wreck, and people who haven't the slightest use for false teeth, wigs, "specs," or hair-dye, can't be old; it is out of the question; they are only growing wise, learning by degrees that it is better to make a friend than an enemy, and that under any circumstances, a man's "last word" should be one of self-respect or courtesy, if not of actual kindness; for then it is the other party who "burns," or passes a sleepless night, not you!

### COURTESY.

No woman can be a lady who would wound or mortify another. No matter how beautiful, how refined, how cultivated she may be, she is in reality coarse, and the innate vulgarity of her nature manifests itself here. Uniformly kind, courteous and polite treatment of all persons, is one mark of a true woman, and of a true man also.—*Anon.*

